# Exploring medication adherence and risk factors in elderly patients with hepatocellular carcinoma undergoing oral targeted therapy: a cross-sectional observational study

**DOI:** 10.3389/fmed.2026.1826926

**Published:** 2026-08-20

**Authors:** Hong Feng, Zhixin Meng, Xian Li

**Affiliations:** Qilu Hospital of Shandong University, Jinan, China

**Keywords:** hepatocellular carcinoma, medication adherence, medication beliefs, risk factors, self-efficacy

## Abstract

**Background:**

Oral targeted therapy is now integral to the management of older adults with hepatocellular carcinoma (HCC), yet suboptimal adherence persistently undermines its real-world effectiveness. Although the Health Belief Model (HBM) is widely applied to explain health behaviors, its integration with self-efficacy theory—particularly in the HCC context, where the volitional gap between belief and action frequently dictates clinical outcomes—remains inadequately characterized.

**Objectives:**

To elucidate factors associated with oral targeted therapy adherence in elderly HCC patients and to evaluate the explanatory utility of an integrated HBM-self-efficacy framework for medication-taking behavior.

**Methods:**

In this cross-sectional study, 653 older HCC patients on oral targeted agents were recruited via convenience sampling. Data were collected using demographic questionnaires, validated HBM and self-efficacy scales, and the Morisky Medication Adherence Scale (MMAS-8). Binary logistic regression and mediation analyses were performed.

**Results:**

The mean MMAS-8 score was 6.08 ± 1.37, with 29.10% of patients classified as having poor adherence. Independent risk factors included moderate-to-severe adverse reactions, high concern beliefs, and living alone, whereas high necessity beliefs and high self-efficacy emerged as protective factors. Notably, self-efficacy partially mediated the relationship between medication beliefs and adherence.

**Conclusion:**

The combined Health Belief Model and self-efficacy framework showed notable ability to explain inter-individual differences in medication adherence within this sample. Medication beliefs and self-efficacy correlated significantly with adherence, while self-efficacy carried a detectable partial indirect association between medication beliefs and adherence.

**Implications for oncology nursing practice:**

Adherence optimization extends beyond initial patient education. Nurses should implement periodic, structured assessments and tailored behavioral interventions—prioritizing the reinforcement of perceived necessity, mitigation of unwarranted concerns, and cultivation of self-efficacy to sustain long-term therapeutic compliance.

## Background

Primary hepatocellular carcinoma (HCC) remains a leading global malignancy, with over 70% of cases diagnosed at advanced stages (1, 2). As the population ages, the proportion of elderly patients affected continues to rise. Advances in molecular biology and precision medicine have positioned oral targeted agents—exemplified by sorafenib and lenvatinib—as a therapeutic mainstay for this demographic, offering enhanced precision and convenience over conventional intravenous chemotherapy (3, 4). Sorafenib, the first oral tyrosine kinase inhibitor (TKI) approved for advanced HCC, has since been joined by lenvatinib, regorafenib, and cabozantinib as first- or second-line options, yet the detailed pharmacodynamics of these agents fall outside the immediate scope of adherence inquiry.

Elderly HCC patients constitute a distinctly vulnerable population whose medication behaviors cannot be extrapolated from general oncology populations. Their adherence is shaped by a complex interplay of physical frailty, cognitive decline, and emotional states—factors that transform daily regimen execution into a substantial behavioral burden. Impaired mobility and memory deficits do not merely increase error rates in the abstract; they render consistent dosing practically arduous. Concurrently, affective disturbances such as fear, anxiety, and depression undermine confidence in therapeutic benefit, gradually eroding the volition to maintain regular intake. Cognitive appraisals are equally decisive: when patients undervalue the survival advantages of targeted therapy while overestimating its side-effect burden, their overall treatment stance skews negatively. Critically, the evidence base specific to this population remains conspicuously thin. Most existing studies on oral targeted therapy adherence in HCC isolate single predictors and operate without a multidimensional, theory-driven analytical framework, leaving the multifactorial determinants of adherence in elderly patients fundamentally under-specified.

To address this gap, we adopted an integrated framework combining the Health Belief Model (HBM)—centered on perceived benefits, barriers, and threats—with Self-Efficacy Theory, the latter being essential for explicating the volitional transition from belief to sustained action (5). Operationally, we utilized the Beliefs about Medicines Questionnaire-Specific (BMQ-Specific), wherein necessity beliefs proxy perceived benefits and concern beliefs proxy perceived barriers, alongside the Rational Medication Self-Efficacy Scale. Employing these measures, we examined medication adherence in elderly HCC patients on oral targeted regimens, with particular focus on how negative emotions and self-efficacy influence adherence outcomes. This investigation aims to inform targeted nursing interventions and render clinical management more precise and systematic.

## Materials and methods

### Study design and setting

This cross-sectional observational study was conducted in the hepatobiliary surgery ward of a tertiary hospital in Shandong Province, China, between August 2024 and August 2025. A convenience sampling method was employed. All 653 participants were hospitalized elderly HCC patients receiving routine inpatient follow-up visits, a setting chosen because it enabled professional nurses to systematically assess adverse reactions per CTCAE criteria, thereby minimizing recall bias inherent in self-reported adverse events. Patients from routine outpatient clinics were not enrolled.

### Participants

Inclusion criteria were: (1) diagnosis of primary HCC conforming to EASL criteria, requiring either pathological confirmation, positive lesions on at least two imaging modalities, or combined imaging findings with alpha-fetoprotein > 400 ng/mL; (2) age ≥ 60 years; (3) Child-Pugh class A or B; (4) receiving oral TKI monotherapy (first- or second-line) and ineligible for or declining guideline-recommended immunotherapy combinations due to clinical or economic barriers; and (5) provision of written informed consent. Exclusion criteria comprised severe mental illness precluding cooperation, or heavy dependence on caregivers for activities of daily living.

### Sample size estimation

Sample size was determined based on the planned binary logistic regression analysis. Sixteen candidate independent variables (13 socio-demographic/clinical indicators and three theory-derived psychological constructs) were pre-specified for potential entry into multivariate models. Applying the events-per-variable (EPV) criterion, a minimum of 160 outcome events was required for EPV = 10, with 320 events recommended for the more stringent EPV = 20. After univariate screening, 12 variables were retained in the final model. With 190 observed poor-adherence events, the effective EPV reached 15.8, exceeding the minimum acceptable threshold. Separately, a priori power analysis for the crude association between self-efficacy and adherence (hypothesized OR = 2.2) indicated that 248 participants would suffice. The final enrollment of 653 participants thus provided robust statistical power for all planned analyses.

### Variables and measurements

Outcome variable: Medication adherence was assessed using the 8-item Morisky Medication Adherence Scale (MMAS-8), with total scores ranging from 0 to 8 and higher scores denoting better adherence (6). For binary logistic regression, adherence was dichotomized using the internationally accepted cut-off: scores < 6 indicated poor adherence, and scores ≥ 6 indicated moderate-to-good adherence.

Core independent variables: Medication beliefs were measured using the Chinese version of Horne’s Beliefs about Medicines Questionnaire-Specific (BMQ-Specific) (7), comprising 5-item Necessity and 5-item Concerns subscales, each scored on a five-point Likert scale (dimension score range 1–5). The Chinese adaptation by Lv et al. (8) demonstrated a content validity of 0.96 and Cronbach’s α of 0.813 (necessity) and 0.706 (concerns). A composite belief score was calculated as necessity minus concerns, with higher scores reflecting stronger overall positive medication beliefs. Medication self-efficacy was evaluated using the 13-item Self-Efficacy for Appropriate Medication Use Scale (SEAMS) (9), scored on a three-point Likert scale, with higher total scores indicating greater self-efficacy (Chinese version Cronbach’s α = 0.934).

Covariates and confounders: Socio-demographic and clinical covariates included age, gender, education level, medical payment status, monthly household income, targeted therapy type, disease duration, number of comorbidities, and living arrangement. Adverse reaction severity was graded by two trained specialist nurses using CTCAE v5.0 via comprehensive chart and laboratory review, with discrepancies resolved by an attending physician. Severity was dichotomized as moderate-to-severe (grade ≥ 2) versus none/mild (grade 0–1) for analysis.

### Data collection and quality control

Data were extracted from three sources: inpatient electronic medical records, outpatient follow-up documentation, and patient-completed questionnaires. All standardized instruments (MMAS-8, BMQ-Specific, and SEAMS) were administered by trained research assistants in a quiet ward setting. Permission for scale usage was obtained from the respective copyright holders. To ensure data integrity, double data entry was performed, and all CTCAE gradings were independently verified by two clinicians prior to final dataset lock.

## Results

### Sample characteristics

Of 682 questionnaires distributed, 29 were excluded due to cognitive impairment, systematic response patterns, or item omission rates ≥ 20%, yielding 653 valid responses (effective response rate 95.75%).

The 653 participants had a mean age of 71.24 ± 5.36 years (range 65–92 years); 492 (75.34%) were male. Educational attainment: primary school or below, 317 (48.55%); junior high school, 206 (31.55%); senior high school or above, 130 (19.91%). Living alone was reported by 142 (.75%), while 511 (78.25%) lived with family. Medical payment: 397 (60.80%) had reimbursement ≥ 80% via medical insurance or New Rural Cooperative Medical Scheme; 256 (39.20%) had reimbursement < 80% or were self-paying. BCLC staging: Stage B, 382 (58.50%); Stage C, 271 (41.50%). Targeted therapy distribution: lenvatinib, 324 (49.62%); sorafenib, 209 (32.01%); donafenib, 120 (18.38%). Medication duration: 6–12 months, 2 (33.84%); ≥12 months, 185 (28.33%). Overall, 471 (72.13%) reported adverse reactions of any grade, with 206 (31.55%) experiencing moderate-to-severe events (CTCAE grade ≥ 2).

### Scale reliability and validity

All instruments demonstrated acceptable psychometric properties in this elderly HCC cohort. The Beliefs about Medicines Questionnaire-Specific (BMQ-Specific) yielded an overall Cronbach’s α of 0.872, with subscale αs ranging from 0.714 to 0.803. Confirmatory factor analysis was consistent with the five-factor structure (χ^2^/df = 2.17, CFI = 0.942, TLI = 0.931, RMSEA = 0.062, SRMR = 0.058). The Self-Efficacy for Appropriate Medication Use Scale (SEAMS) showed a total Cronbach’s α of 0.846 (subscale range 0.702–0.795). The MMAS-8 had a Cronbach’s α of 0.763 and a criterion-related validity coefficient of 0.624 (*P* < 0.001) against medication possession rate.

### Distribution of core variables

The mean MMAS-8 total score was 6.08 ± 1.37. Adherence distribution: good (score ≥ 6), 4 (32.77%); moderate, 249 (38.13%); poor (score < 6), 190 (29.10%). To mitigate potential misclassification near the dichotomization threshold, a sensitivity analysis restricted to the 5–7 score band (near the cut-off) was performed (Supplementary Table 1). In this subgroup, the direction, magnitude, and statistical significance of associations for all key predictors remained consistent with the primary analysis, confirming the robustness of the binary classification.

BMQ-Specific subscale scores (range 1–5) were: necessity belief, 4. ± 0.58; concern belief, 2.89 ± 0.81. The SEAMS total score was 3.24 ± 0.76, indicating a moderate level of self-efficacy.

### Univariate analysis

Chi-square tests revealed significant differences in adherence across categories of age, education, living arrangement, medical payment, medication duration, adverse reaction severity, and number of comorbidities (all *P* < 0.05). Specifically, patients aged ≥ 75 years, with primary education or below, living alone, with reimbursement < 80% or self-paying, on medication ≥ 1 year, with moderate-to-severe adverse reactions, or having ≥3 chronic comorbidities exhibited higher proportions of poor adherence (Table 1).

**TABLE 1:** Univariate analysis of demographic and clinical variables.

Variable category	Variable	Subgroup classification	Total cases	Good/moderate adherence (*n* = 463)	Poor adherence (*n* = 190)	χ ^2^ value	*P*-value
Demographic characteristics	Age (year)	65∼74	412	314 (76.21)	98 (23.79)	15.72	<0.001***
		≥75	241	149 (61.83)	92 (38.17)	–	–
	Sex	Male	492	347 (70.53)	145 (29.47)	0.37	0.542
		Female	161	116 (72.05)	45 (27.95)	–	–
	Degree of education	Primary school and below	317	196 (61.83)	1 (38.17)	36.18	<0.001***
		Junior middle school	206	154 (74.76)	52 (25.24)	–	–
		High school or above	130	113 (86.92)	17 (13.08)	–	–
	The resident manner	Living alone	142	70 (49.30)	72 (50.70)	47.96	<0.001***
		Living with family	511	393 (76.91)	118 (23.09)	–	–
	Domicile	City	333	9 (65.77)	114 (34.23)	1.43	0.4
		Countryside	320	248 (77.50)	72 (22.50)	–	–
	Primary caregiver	Spouse	320	226 (70.63)	94 (29.37)	1.33	0.511
		Children	201	145 (72.14)	56 (27.86)	–	–
		Housekeeper	59	44 (74.58)	15 (25.42)	–	–
		Oneself	73	48 (65.75)	25 (34.25)	–	–
	Monthly per capita household income (yuan)	<3,000	258	156 (60.47)	102 (39.53)	28.54	<0.001***
		3,000∼<5,000	241	179 (74.27)	62 (25.73)	–	–
		≥5000	154	128 (83.12)	26 (16.88)	–	–
	Medical payment methods	Medical insurance reimbursement rate ≥ 80%	397	312 (78.59)	85 (0.41)	26.97	<0.001***
		Reimbursement rate < 80%/ out-of-pocket	256	151 (58.98)	105 (41.02)	–	–
Clinical features	BCLC by stages	B designated time	382	284 (74.35)	98 (25.65)	4.83	0.028*
		C designated time	271	179 (66.05)	92 (33.95)	–	–
	Types of targeted drugs	Lenvatinib	324	228 (70.37)	96 (29.63)	1.29	0.523
		Sorafenib	209	150 (71.77)	59 (28.23)	–	–
		Donafenib	120	85 (70.83)	35 (29.17)	–	–
	Duration of medication	<6 months	247	199 (80.57)	48 (19.43)	32.66	<0.001***
		6∼<12 months	2	159 (71.95)	62 (28.05)	–	–
		≥12 months	185	105 (56.76)	80 (43.24)	–	–
	Number of comorbidities	0∼1 species	226	188 (83.19)	38 (16.81)	58.27	<0.001***
		2∼3 species	307	220 (71.66)	87 (28.34)	–	–
		≥4 species	120	55 (45.83)	65 (54.17)	–	–
	Adverse drug reaction severity	Not mild	447	371 (83.00)	76 (17.00)	102.13	<0.001***
		Moderate to severe	206	92 (44.66)	114 (55.34)	–	–

* Indicates *P* < 0.05,

*** indicates *P* < 0.001.

### Correlation analysis

Pearson correlation analysis showed that MMAS-8 scores were significantly positively correlated with perceived necessity beliefs (*r* = 0.382, *P* < 0.001) and self-efficacy (*r* = 0.453, *P* < 0.001), and significantly negatively correlated with perceived concerns (*r* = −0.417, *P* < 0.001).

### Multivariate logistic regression

Binary logistic regression was performed with medication adherence as the dependent variable (0 = moderate-to-good adherence, 1 = poor adherence). Independent variables entering the model were the 12 factors that reached statistical significance in univariate analysis: age, education, living arrangement, per capita monthly household income, medical payment method, BCLC stage, medication duration, adverse reaction severity, number of chronic comorbidities, and the three core psychological constructs (necessity belief, concern belief, and self-efficacy). All continuous predictors were standardized to Z-scores prior to entry. Collinearity diagnostics indicated no substantial multicollinearity (all VIF < 3).

The final model was statistically significant (omnibus χ^2^ = 227.46, *P* < 0.001) and demonstrated good fit (Hosmer-Lemeshow χ^2^ = 8.26, *P* = 0.408). Detailed regression coefficients are presented in Table 3.

### Explanatory power of the integrated framework

A multiple linear regression model incorporating all medication belief dimensions and self-efficacy yielded an adjusted R^2^ of 0.482 for MMAS-8 total score, indicating that the integrated framework explained 48.2% of adherence variance. By comparison, the model containing medication beliefs alone achieved an adjusted R^2^ of 0.327, and beliefs-only without self-efficacy yielded 0.206. The inclusion of self-efficacy thus improved explanatory power by 32.2%, supporting the added value of the composite framework.

### Mediation analysis

Mediation was tested using Hayes’ PROCESS macro (Model 4) with 5,000 bootstrap resamples and bias-corrected 95% confidence intervals, adjusting for age, living arrangement, adverse reaction severity, and medication duration.

**FIGURE 1:**
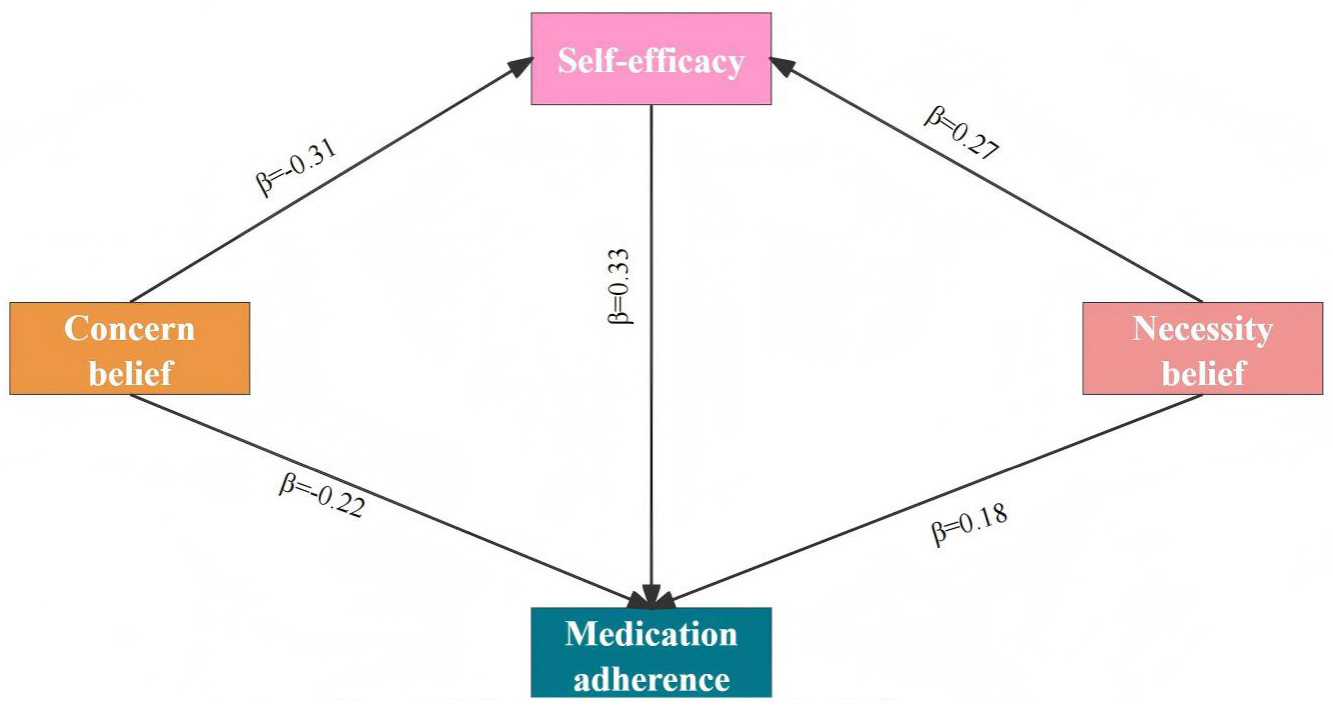
Mediating effect path analysis of self-efficacy.

For the concern belief → adherence pathway, the total effect was -0.319 (95% CI: −0.397 to −0.241, *P* < 0.001), with a direct effect of −0.7 (95% CI: −0.289 to −0.145, *P* < 0.001) and an indirect effect via self-efficacy of −0.102 (95% CI: −0.146 to −0.063), accounting for 31.97% of the total effect. For the necessity belief → adherence pathway, the total effect was 0.271 (95% CI: 0.194–0.348, *P* < 0.001), direct effect 0.184 (95% CI: 0.112–0.256, *P* < 0.001), and indirect effect 0.087 (95% CI: 0.049–0.131), representing 32.10% of the total effect. Standardized path coefficients (Figure 1) were β = 0.27 for necessity belief → self-efficacy, and β = 0.33 for self-efficacy → adherence (Figure 1 and Table 2).

**TABLE 2:** Mediating effect test of self-efficacy between health belief dimensions and oral targeted medication adherence.

Pathway	Effect size	Boot SE	95% CI	Effect proportion
Concern belief → medication adherence	–	–	–	–
Gross effect	−0.319	0.040	−0.397∼-0.241	100%
Direct effect	−0.7	0.037	−0.289∼-0.145	68.03%
Mediation effect (self-efficacy)	−0.102	0.0	−0.146∼-0.063	31.97%
Necessity belief → medication adherence	–	–	–	–
Gross effect	0.271	0.039	0.194∼0.348	100%
Direct effect	0.184	0.037	0.112∼0.256	67.90%
Mediation effect (self-efficacy)	0.087	0.0	0.049∼0.131	32.10%

### Independent predictors of non-adherence

Binary logistic regression identified independent predictors of poor adherence (Table 3). Moderate-to-severe adverse reactions emerged as a significant risk factor (OR = 2.31, 95% CI: 1.42–3.76, *P* < 0.001). Protective factors included higher necessity belief (OR = 0.68, 95% CI: 0.53–0.87, *P* = 0.002) and stronger self-efficacy (OR = 0.72, 95% CI: 0.57–0.91, *P* = 0.004), whereas elevated concern belief independently increased non-adherence risk (OR = 1.82, 95% CI: 1.29–2.57, *P* < 0.001). All Wald statistics were statistically significant.

**TABLE 3:** Significant independent correlates of medication non-adherence from adjusted binary logistic regression.

Variable	Regression coefficient β	Wald χ ^2^	*P*-value	Odds ratio (OR)	95% confidence interval of OR
Adverse drug reactions	0.837	11.25	<0.001	2.31	1.42–3.76
Necessity belief	−0.386	9.87	0.002	0.68	0.53–0.87
Concern belief	0.599	13.62	<0.001	1.82	1.29–2.57
Self-efficacy	−0.329	8.44	0.004	0.72	0.57–0.91

The multivariate model simultaneously adjusted for eight confounding variables (age, educational level, living arrangement, household monthly income, medical reimbursement status, BCLC stage, oral targeted therapy duration, number of chronic comorbidities). Full regression results for all 12 modeled variables are provided in Supplementary Table 2. No statistically significant independent associations were detected for the eight adjusted covariates (all P > 0.05), and no suppression effects were identified.

### Chain mediation analysis

**FIGURE 2:**
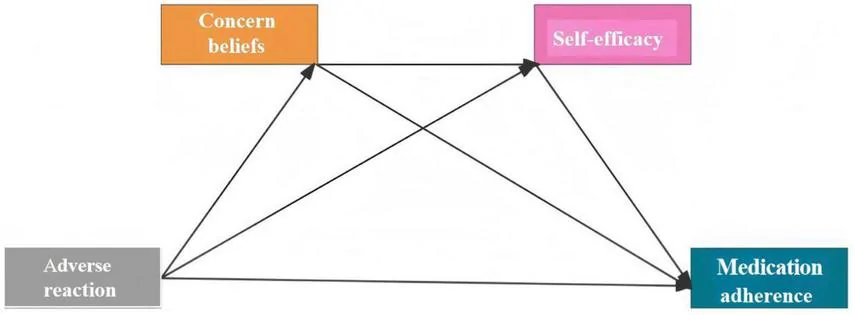
Path diagram showing serial correlational associations across adverse reactions, concern beliefs, self-and medication adherence.

Serial mediation (PROCESS Model 6) tested the pathway: adverse reaction severity → concern belief → self-efficacy → adherence. The indirect effect along this pathway was −0.074 (95% CI: −0.112, −0.041), accounting for 22.7% of the total effect. Greater adverse reaction severity was associated with heightened concern beliefs, which were further associated with reduced self-efficacy, and this in turn with lower adherence (Figure 2 and Table 4).

**TABLE 4:** Chain mediation effect analysis.

Pathway	Effect size	Boot SE	95% CI	Effect proportion
Gross effect	−0.326	0.042	−0.408∼−0.244	100%
Direct effect	−0.152	0.039	−0.229∼−0.075	46.60%
Total indirect effect	−0.174	0.028	−0.231∼−0.1	53.40%
Specific path				
Adverse reactions → concern beliefs →medication adherence	−0.068	0.018	−0.105∼−0.035	20.90%
Adverse reactions → self-efficacy → medication adherence	−0.032	0.012	−0.058∼−0.011	9.80%
Adverse reactions → concern beliefs → self-efficacy → medication adherence	−0.074	0.018	−0.112∼−0.041	22.70%

## Discussion

### Epidemiological and clinical context

The 2024 national cancer burden report (10) ranks HCC fifth in incidence and second in cancer-related mortality in China. While the 45–59-year age group carries the highest new-case burden, the 60–79-year cohort accounts for 157,390 new cases, indicating that a substantial number of elderly patients (≥65 years) are routinely identified in clinical practice.

Consistent with Pan-Asian ESMO-CSCO guidelines (11), immune checkpoint inhibitor–anti-angiogenic combinations are the preferred first-line therapy for eligible unresectable HCC (12, 13). Oral TKIs remain a guideline-endorsed alternative for patients with immunotherapy exclusion criteria, including active autoimmune disease, high variceal bleeding risk, borderline Child-Pugh status, poor ECOG performance, or financial constraints. Our cohort (August 2024–August 2025) enrolled 653 patients aged ≥ 65 years on TKI monotherapy, as most screened candidates did not meet immunotherapy eligibility criteria. All participants were hospitalized for routine follow-up rather than community-dwelling outpatients; this sampling approach may limit generalizability to all community-dwelling elderly HCC patients on oral targeted agents.

Consistent adherence to oral TKI regimens has been associated with the survival outcomes reported in pivotal trials such as REFLECT (4, 14). Notably, these trials did not conduct adherence-stratified subgroup analyses. The crude adherence–survival association is subject to confounding by indication: patients with better tolerance, milder adverse events, and superior baseline reserve tend to sustain treatment and experience longer survival, rather than adherence independently conferring a fixed survival benefit. Nonetheless, real-world data indicate that only 48.4%–58.89% of elderly cancer patients maintain adequate adherence to long-term oral targeted therapy (15, 16).

Prior research has largely examined isolated adherence determinants without elucidating underlying mechanisms. The present study integrates the HBM with self-efficacy theory in a cohort of 653 older HCC adults; the model explained 48.2% of adherence variance, providing a theoretical basis for targeted intervention development.

### Current status of adherence and clinical implications

The mean MMAS-8 score was 6.08 ± 1.37, with only 32.77% demonstrating good adherence. Nearly 30% reported missed doses or unilateral treatment adjustment, aligning with prior Chinese cohort studies (17, 18) and highlighting an unmet need in adherence management.

Patients with treatment duration ≥12 months had a poor adherence rate of 43.24%, significantly higher than those on therapy <6 months. This pattern may reflect treatment fatigue associated with long-term medication (19), cumulative adverse reactions, and reduced follow-up intensity. Current clinical practice often emphasizes adherence at treatment initiation but lacks longitudinal support. These findings suggest that adherence management should span the entire treatment course, with assessment and supportive interventions every 1–2 months for patients beyond 12 months of therapy.

No significant adherence differences were observed across targeted agents (lenvatinib, sorafenib, donafenib; *P* = 0.523), consistent with their comparable once-daily oral administration and adverse-effect profiles. Accordingly, adherence interventions may not require drug-specific tailoring; standardized, broadly applicable tools may suffice.

### Factors associated with adherence

Multivariable analysis identified four factors associated with poor adherence: (1) moderate-to-severe adverse reactions, (2) living alone, (3) stronger medication concerns, and (4) treatment duration ≥ 12 months. Stronger necessity beliefs and higher self-efficacy emerged as protective correlates.

Adverse reactions showed the strongest association with poor adherence (OR = 2.31, 95% CI: 1.42–3.76, *P* < 0.001). Older adults have reduced physiological reserve and may tolerate toxicities such as hand–foot skin reaction, diarrhea, and fatigue less well. These symptoms are frequently misinterpreted as disease progression or severe toxicity, prompting unsupervised dose reduction or discontinuation (20). Among patients with moderate-to-severe reactions, 55.34% showed poor adherence. This finding supports proactive, systematic adverse-event management—including pre-treatment education on expected toxicities and self-management strategies, and regular monitoring with dose adjustments or supportive care as needed.

Health beliefs were core adherence correlates: each 1-SD increase in necessity beliefs was associated with a 32% lower likelihood of poor adherence (OR = 0.68, 95% CI: 0.53–0.87, *P* = 0.002); each 1-SD increase in concern beliefs was associated with an 82% higher likelihood (OR = 1.82, 95% CI: 1.29–2.57, *P* < 0.001). These findings align with the HBM, highlighting the opposing associations of perceived benefits and barriers with adherence (21). The most frequently endorsed concerns were: (1) high drug costs and perceived financial burden on family; (2) apprehension that adverse effects would be intolerable; and (3) fear of forgetting doses. Adherence education should address these specific barriers—clarifying reimbursement policies and assistance programs, and communicating in accessible language that most adverse effects are manageable and reversible.

Self-efficacy was associated with lower odds of poor adherence (OR = 0.72, 95% CI: 0.57–0.91, *P* = 0.004). Patients living alone had self-efficacy scores 0.68 points lower than those living with family, suggesting that reduced social support may correlate with lower confidence in self-management. Clinically, self-efficacy may be strengthened through family or caregiver engagement, reminder systems, pill organizers, and mobile alarms.

### Explanatory value of the integrated framework

The integrated HBM–self-efficacy model explained 48.2% of adherence variance, substantially exceeding the standalone HBM (adjusted R^2^ = 0.327). This supports the added value of incorporating self-efficacy to address the belief–behavior gap commonly observed in HBM applications.

Mediation analyses indicated that self-efficacy partially mediated the associations between health beliefs and adherence, with indirect effects accounting for approximately 32% of the respective total effects. This observed pattern is consistent with Bandura’s self-efficacy theory (22). However, given the cross-sectional design, alternative directional associations are equally plausible: low self-efficacy may correlate with negative health beliefs, and poor adherence may correlate with negative reappraisal of medication benefits and risks.

Chain mediation (PROCESS Model 6) quantified the serial pathway: adverse reaction severity → concern belief → self-efficacy → adherence. The indirect effect was −0.074 (95% CI: −0.112, −0.041), accounting for 22.7% of the total effect. This observed correlational pattern is consistent with a hypothesized sequential linkage—greater adverse reaction severity was associated with heightened concerns, which were further associated with reduced self-efficacy, and this in turn with lower adherence—but does not confirm temporal causality or directional mechanisms. Clinically, this pattern supports proactive adverse-event management and cognitive intervention strategies that may help interrupt this potential chain. Self-efficacy, as the terminal and most actionable link, may be strengthened via graded goal setting, skills training, performance feedback, and peer support.

Collectively, these findings offer preliminary evidence for the potential utility of the HBM–self-efficacy framework in understanding medication adherence among older adults—a population that has been underrepresented in previous HBM-based adherence research. The identified obstacles, including cognitive decline, increased dependence on family support, and reduced decision-making confidence, underscore the particular relevance of these psychological constructs in this age group. Furthermore, the observed mediating role of self-efficacy suggests that incorporating this construct may contribute to a more nuanced understanding of adherence behaviors in geriatric oncology, with possible implications for adherence research in other chronic conditions such as hypertension and diabetes.

### Comparison with previous studies

A systematic review of prior HCC adherence studies (23–25) revealed common limitations: they either compared survival outcomes across patient assistance programs, screened demographic risk factors without psychological theory, or relied solely on insurance claims lacking clinical liver disease indicators. None integrated HBM with self-efficacy in a unified framework, focused specifically on elderly HCC, or applied bootstrap-based mediation decomposition to quantify sequential correlational pathways.

The present study addresses these gaps through four distinct strengths: (1) an integrated HBM–self-efficacy theoretical framework; (2) inclusion of objective clinical liver disease indicators; (3) a focus on the clinically vulnerable elderly HCC subgroup; and (4) actionable nursing intervention strategies informed by PROCESS-based mediation analysis. This multi-dimensional design distinguishes our work from previously published observational research.

### Limitations and future directions

Several limitations should be acknowledged. First, all mediation analyses were based on cross-sectional data with simultaneous variable measurement. The absence of temporal precedence precludes causal inference; all observed path associations reflect statistical correlations in the current sample, and alternative directional pathways are equally plausible. Prospective longitudinal studies with staggered measurement are required to verify directional relationships.

Second, eligibility criteria may have introduced selection bias. Patients with Child–Pugh class C, advanced dementia, or severe functional dependence were excluded, limiting generalizability to those with Child–Pugh A–B status, intact cognition, and basic ADL independence.

Third, adherence was primarily assessed via self-report, supplemented by MPR verification. While this provides pragmatic triangulation, recall and social-desirability biases remain possible, particularly in older adults.

Fourth, all participants were recruited from hospitalized wards rather than stable outpatients. Hospitalized patients may have interrupted or newly initiated medication, leading to systematic differences compared with community outpatients. Mixed inpatient–outpatient sampling is recommended for future research.

Future work should consider incorporating additional HBM constructs—particularly external cues to action (e.g., caregiver reminders, digital alerts)—to further refine the model. Multi-center designs with larger sample sizes would improve generalizability. Methodologically, combined adherence assessment strategies integrating pharmacy dispensing records, electronic pill containers, or app-based logs would reduce reliance on subjective reporting and provide more robust evidence for standardized medication management in older HCC patients.

## Data Availability

The original contributions presented in this study are included in this article/Supplementary material, further inquiries can be directed to the corresponding author.
