# Supplementary material for: Exploring medication adherence and risk factors in elderly patients with hepatocellular carcinoma undergoing oral targeted therapy: a cross-sectional observational study
Source: Front Med (Lausanne). 2026 Aug 20;13:1826926. doi: 10.3389/fmed.2026.1826926 (PMC13539135; doi:10.3389/fmed.2026.1826926)
Supplement: Supplementary file 1 [file Table_1.docx]

**Supplementary Table 1**

To test whether the dense distribution of participants near the MMAS-8 cutoff of 6 biased our main findings, a sensitivity analysis was conducted by restricting the sample to patients with MMAS-8 scores ranging from 5 to 7 (n=407). Fully adjusted binary logistic regression with consistent covariates was repeated in this subgroup, and complete outputs are presented in Supplementary Table S1. All predictors with significant crude associations identified via univariate chi-square tests remained statistically independent risk factors for poor adherence in the restricted subgroup. Adjusted odds ratios exhibited mild attenuation but consistent effect directions and overlapping 95% confidence intervals between the full cohort and narrow-score subgroup. No variable lost statistical significance or reversed risk magnitude, confirming the robustness of our multivariate results against minor shifts around the MMAS-8 dichotomization threshold.

**Supplementary Table 1 Sensitivity analysis was conducted on patients (n=407) with MMAS-8 scores between 5 and 7**

| **Independent Variables (Reference Group)** | **Full Sample (N=653)** |  | **Restricted Subgroup (MMAS-8=5–7, N=407)** |  |
| --- | --- | --- | --- | --- |
|  | **Adjusted OR (95%CI)** | **P-value** | **Adjusted OR (95%CI)** | **P-value** |
| **Demographic Characteristics** |  |  |  |  |
| Age ≥75 years (ref: 65–74 years) | 1.73 (1.21–2.48) | 0.003** | 1.68 (1.12–2.52) | 0.012* |
| Primary school and below (ref: High school or above) | 2.01 (1.38–2.92) | <0.001*** | 1.95 (1.27–3.00) | 0.002** |
| Junior middle school (ref: High school or above) | 1.52 (1.03–2.24) | 0.035* | 1.48 (0.97–2.26) | 0.045* |
| Living alone (ref: Living with family) | 2.26 (1.54–3.31) | <0.001*** | 2.18 (1.43–3.33) | <0.001*** |
| Monthly per capita household income <3000 yuan (ref: ≥5000 yuan) | 1.86 (1.29–2.67) | 0.001** | 1.81 (1.20–2.73) | 0.005** |
| Monthly per capita household income 3000~<5000 yuan (ref: ≥5000 yuan) | 1.34 (0.92–1.95) | 0.128 | 1.30 (0.86–1.97) | 0.211 |
| Medical insurance reimbursement rate <80% / out-of-pocket (ref: ≥80%) | 2.19 (1.51–3.17) | <0.001*** | 2.11 (1.40–3.19) | <0.001*** |
| Female (ref: Male) | 1.07 (0.71–1.61) | 0.753 | 1.04 (0.66–1.65) | 0.862 |
| Rural domicile (ref: City) | 0.93 (0.65–1.33) | 0.689 | 0.91 (0.61–1.36) | 0.645 |
| Primary caregiver: Children (ref: Spouse) | 0.95 (0.64–1.41) | 0.793 | 0.93 (0.60–1.44) | 0.748 |
| Primary caregiver: Housekeeper (ref: Spouse) | 0.90 (0.51–1.58) | 0.714 | 0.88 (0.47–1.64) | 0.687 |
| Primary caregiver: Oneself (ref: Spouse) | 1.12 (0.68–1.84) | 0.66 | 1.10 (0.63–1.92) | 0.732 |
| **Clinical Characteristics** |  |  |  |  |
| BCLC stage C (ref: BCLC stage B) | 1.49 (1.05–2.11) | 0.024* | 1.45 (1.00–2.10) | 0.048* |
| Medication duration <6 months (ref: ≥12 months) | 2.14 (1.47–3.12) | <0.001*** | 2.08 (1.36–3.18) | <0.001*** |
| Medication duration 6~<12 months (ref: ≥12 months) | 1.58 (1.09–2.29) | 0.016* | 1.54 (1.02–2.32) | 0.041* |
| Number of comorbidities ≥4 species (ref: 0~1 species) | 2.37 (1.64–3.42) | <0.001*** | 2.29 (1.52–3.46) | <0.001*** |
| Number of comorbidities 2~3 species (ref: 0~1 species) | 1.62 (1.14–2.30) | 0.007** | 1.57 (1.06–2.33) | 0.024* |
| Moderate to severe adverse drug reactions (ref: Mild / no reaction) | 2.45 (1.70–3.54) | <0.001*** | 2.36 (1.57–3.55) | <0.001*** |
| Targeted drug type: Sorafenib (ref: Lenvatinib) | 0.96 (0.66–1.39) | 0.82 | 0.94 (0.62–1.42) | 0.766 |
| Targeted drug type: Donafenib (ref: Lenvatinib) | 0.98 (0.64–1.49) | 0.915 | 0.95 (0.60–1.51) | 0.824 |

| **Variable** | **β** | **Wald χ²** | **P** | **OR** | **95% CI for OR** |
| --- | --- | --- | --- | --- | --- |
| Adverse drug reactions | 0.837 | 11.25 | <0.001 | 2.31 | 1.42–3.76 |
| Necessity belief | -0.386 | 9.87 | 0.002 | 0.68 | 0.53–0.87 |
| Concern belief | 0.599 | 13.62 | <0.001 | 1.82 | 1.29-2.57 |
| Self-efficacy | -0.329 | 8.44 | 0.004 | 0.72 | 0.57–0.91 |
| Age | 0.143 | 1.26 | 0.261 | 1.15 | 0.90–1.47 |
| Education | 0.121 | 0.93 | 0.334 | 1.13 | 0.88–1.45 |
| Primary caregiver | 0.168 | 1.51 | 0.219 | 1.18 | 0.91–1.53 |
| Monthly per capita household income (yuan) | 0.105 | 0.78 | 0.377 | 1.11 | 0.87–1.42 |
| Medical payment methods | 0.134 | 1.12 | 0.290 | 1.14 | 0.89–1.46 |
| BCLC by stages | 0.096 | 0.65 | 0.420 | 1.10 | 0.86–1.41 |
| Duration of medication | 0.152 | 1.38 | 0.240 | 1.16 | 0.90–1.49 |
| Number of comorbidities | 0.117 | 0.86 | 0.354 | 1.12 | 0.88–1.43 |

**Supplementary Table 2 Binary Logistic Regression Analysis of Independent Predictors for Medication Non-Adherence**
